# The Role of Chemosensory Proteins in Insecticide Resistance: A Review

**DOI:** 10.3390/insects16050496

**Published:** 2025-05-05

**Authors:** Angeliki Tsouri, Vassilis Douris

**Affiliations:** 1 Department of Biological Applications and Technology, University of Ioannina, 45110 Ioannina, Greece; ang.tsouri@uoi.gr; 2 Biomedical Research Institute—Foundation for Research and Technology—Hellas (BRI-FORTH), 45110 Ioannina, Greece

**Keywords:** chemosensory proteins, insecticide resistance, resistance mechanisms, synergistic interactions

## Abstract

Chemosensory proteins (CSPs) are small soluble proteins found predominantly in insects, with a conserved structure that contains a hydrophobic cavity. While originally associated with chemosensation, they were soon implicated to several other functions related to their ability to bind hydrophobic molecules. Research in the last decade has shown that CSPs may play a role in insecticide resistance. Several *CSP* genes are upregulated upon induction by sublethal insecticide doses or are highly expressed in resistant populations. RNA interference of *CSP* genes can restore susceptibility to insecticides. In vitro binding assays and molecular docking simulations suggest that CSPs can strongly bind to insecticides and can accommodate even large molecules in their hydrophobic cavities. Some cases of *CSP* overexpression in transgenic insects conferring insecticide resistance are reported. Taken together, these results indicate a role for CSPs in insecticide resistance, presumably through a sequestration mechanism, perhaps in combination with other mechanisms like metabolic resistance. This article reviews the evidence for CSP involvement in resistance and discusses ongoing research in the field.

## Simple Summary

Insects can be either friends or foes regarding food security and human health. To protect harvests or prevent diseases, insect populations must be controlled. This frequently requires the use of chemical insecticides. However, insects can develop resistance to insecticides via several molecular mechanisms, posing significant challenges for pest control. Several insect proteins may take part in resistance. One type of protein that may be involved are chemosensory proteins (CSPs). While originally believed to have functions related to sensing the environment and releasing signals such as pheromones, CSPs are now considered key players in so-far-unrecognized mechanisms of insecticide resistance. This article reviews the evidence for CSP involvement in resistance and discusses ongoing research in the field.

## 1. Introduction

Chemosensory proteins (CSPs) are a class of small soluble proteins, specific to arthropods and most notably to insects [1,2,3,4,5]. First reported in cockroaches [6] and identified in *Drosophila melanogaster* [7], they were later found to be expressed in the sensillar lymph of several insects and a chemodetection function was implicated for the whole family [1], presumably in a manner analogous to the well-studied Odorant Binding Protein (OBP) family. However, several other functions have been postulated for CSPs, unrelated to chemosensation. Given their small size, compact but flexible structure, and ability to carry hydrophobic compounds, they have been implicated in pheromone storage and delivery, nutrient solubilization, development, regeneration, and, more recently, insecticide resistance (reviewed in [5]). Their potential role in insecticide resistance is discussed in this review.

Similar to OBPs, CSPs are small (c. 13 kDa) proteins, with *α*-helical domains that form a hydrophobic cavity [8,9,10,11]. While OBP structure is maintained by three disulfide bridges, in CSPs, there is a conserved motif of 4 cysteines, the first pair separated by 6–8 residues and the second pair by 2 residues, each pair connected by disulfide bridges joining adjacent cysteines [1]. A handful of CSP structures have been experimentally defined so far [8,11,12,13,14].

CSP amino acid sequences are quite conserved compared to OBPs, often exhibiting 40–50% identical residues between orthologues from distant species [3,5]. Presumably, the three interlocked disulfide bonds in OBPs contribute to a stable and conserved structure of the protein, while in CSPs, there must be higher sequence conservation to maintain folding. On the other hand, this makes CSPs more flexible than OBPs, which implicates a larger range of sizes and shapes of potential ligands than most OBPs [5]. Interestingly, significant conformational modifications have been reported for CSPs upon binding, enabling them to accommodate quite large ligands [8,15]. Thus, CSPs can apparently enlarge their binding cavities since the two disulfide bridges do not hinder the change in conformation.

While *CSP* genes within eucaryotic genomes appear arthropod-specific (though similar sequences are found in bacteria [16]), only one or two gene copies are present in Chelicerata, Crustacea and Myriapoda, a number “*too limited to support an important function in chemical detection*” [5]. However, within the Hexapoda, the *CSP* family seems to have expanded significantly [3,4,5] with variable number of genes, from 3-4 genes in *Drosophila* [17], 8 in *Anopheles* mosquitoes [3], 6 in the honeybee *Apis mellifera* [18], 15 in *Nilaparvata lugens* [19], and 27 in *Helicoverpa armigera* [20] to as many as 70 in *Locusta migratoria* [21]. It is possible that this expansion has enabled differentiation of the several functions CSPs are implicated in within insect species. An additional factor that could possibly further enhance CSP diversity is a presumed RNA editing mechanism [16].

## 2. CSPs Have Been Implicated to Several Different Functions

The first report of a soluble protein that was later recognized as a CSP was described in relation to limb regeneration in the cockroach *Periplaneta americana* [6,22]. Later research has shown that many CSPs are abundant in tissues related to chemodetection (e.g., antennae, legs, palps, proboscis, etc.) in several insect orders. Thus, they were considered as another class of binding proteins along with OBPs [23]. This was supported also by the ability of certain CSPs to bind semiochemicals with μΜ dissociation constants, similarly to OBPs [24]. Several recent studies indicate that *CSPs* expressed in chemosensory organs play a crucial role in detecting pheromones and plant odors [25,26,27,28,29,30,31], though this research topic is beyond the scope of this review.

On the other hand, *CSP* expression has been identified in several non-chemosensory organs (e.g., heads, thorax, midgut, fat body, ovary, cuticle, and reproductive organs) [5,20,21,32,33,34]. The presence of certain CSPs in pheromone glands led to the suggestions that CSPs can also act as solubilizers of hydrophobic pheromones produced by the glands and facilitate their release [35]. A role for CSPs is postulated regarding several behavioral functions, including searching for hosts, egg laying, and mating. Knockdown of a *CSP* gene led to a disruption in soil-guided behavior in *Hylamorpha elegans* [36], while numerous recent studies have indicated that there might be a connection between CSPs and reproductive processes. For instance, silencing of *BtabCSP11* of the whitefly *Bemisia tabaci* led to decreased fecundity [37], while RNAi of *CSP12* in the leaf beetle *Ophraella communa* reduced the number of eggs [38]. The absence of *AlepCSP2* in *Athetis lepigone* results in a reduced mating rate [30]. Several CSPs in *Spodoptera litura* are associated with reproduction [39]. *CSP20* from *Spodoptera exigua* is highly expressed in the testes and probably plays a role in sperm and testis development, while other CSPs may have dual roles both in chemoreception and in reproductive physiology [40]. Other CSPs that may be related to mating and reproduction are reported in *B. tabaci* [33] and *Dioryctria abietella* [29].

Some reports implicate CSPs with specific developmental processes; *CSP5* of the honeybee is expressed exclusively in the ovaries and eggs, but when the gene is silenced, embryonic development and egg hatching was prevented [32,41]. Similarly, silencing of *CSP9* of the red fire ant *Solenopsis invicta* interferes with fatty acid biosynthesis and prevents cuticle development and ecdysis [42]. Nevertheless, it is possible that these actions are not mediated by the CSP itself but by specific ligands bound to the protein, such as hormones [5]. Other studies implicate CSPs in nutrition as solubilizers and carriers of hydrophobic nutrients and/or surfactants in the proboscis to reduce pressure during sucking [15,43]. A similar role was proposed for CSPs as carriers for hydrophobic compounds (pigments) required for vision [15].

Several lines of evidence point to a major role for CSPs within the context of insecticide resistance. This review will further below focus on aspects of this emerging research field.

## 3. Evidence for an Insecticide Resistance Function of CSPs

### 3.1. Gene Expression

#### 3.1.1. Certain *CSPs* Are Upregulated Following Insecticide Treatment

The first evidence associating CSPs with insecticide resistance came from the observation that some *CSP* genes are upregulated following insecticide treatment (indicated in detail in Table 1). This was shown initially in *B. tabaci* in response to the neonicotinoid thiamethoxam [44,45] and in two Lepidopterans, the silkmoth *Bombyx mori* following treatment with avermectins [46] and the diamondback moth, *Plutella xylostella* after exposure to the pyrethroid permethrin [47].

Several subsequent studies involving different insect species and compound chemistries have provided similar results (Table 1). Quantitative PCR analysis of mRNA levels of eight *CSPs* of the aphid *Rhopalosiphum padi* showed that they were upregulated after imidacloprid and β-cypermethrin treatments [48], while in the same species, the expression levels of five *CSPs* were upregulated after exposure to thiamethoxam [49] and three *CSPs* after exposure to deltamethrin [50]. Expression of at least one *CSP* was induced following treatment with flubendiamide in *Ostrinia furnacalis* [51], while several *CSP* genes were significantly upregulated following induction by λ-cyalothrin in the litchi fruit borer *Conopomorpha sinensis* [52], indoxacarb in *P. xylostella* [53], dichlorvos and carbofuran in *Tribolium castaneum* [54], chlorpyrifos, emamectin benzoate, and fipronil in *S. litura* [55], and deltamethrin in *Anopheles gambiae* [56]. In the cotton aphid *Aphis gossypii*, some *CSPs* were upregulated following treatment with omethoate in a dose-dependent manner [57]. Multiple insecticides (spinetoram, chlorantraniliprole, cypermethrin, chlorfenapyr, chlorpyrifos, and indoxacarb) induce upregulation of two cuticular *CSPs* in *Spodoptera frugiperda* [34].

**Table 1:** Details of chemosensory protein genes upregulated following insecticide treatment.

CSP Name	Organism	Insecticide (s)	Reference
*BtabCSP1*	*Bemisia tabaci*	Thiamethoxam	[44,45]
*SAP2*, *CSP6*, *SAP3*, *CSP4*	*Anopheles gambiae*	Deltamethrin	[56]
*DcitCSP8*	*Diaphorina citri*	Thiamethoxam	[58]
*TcCSP10*	*Tribolium castaneum*	Dichlorvos, Carbofuran	[54]
*RpCSP1*, *RpCSP2*, *RpCSP4*, *RpCSP5*, *RpCSP6*, *RpCSP7*, *RpCSP8*, *RpCSP10*	*Rhopalosiphum padi*	Imidacloprid, β-cypermethrin	[48]
*RpCSP1*, *RpCSP5*, *RpCSP7 RpCSP4*, *RpCSP10*	*Rhopalosiphum padi*	Thiamethoxam	[49]
*RpCSP6*, *RpCSP7*, *RpCSP8*	*Rhopalosiphum padi*	Deltamethrin	[50]
*CsCSP1*, *CsCSP2*, *CsCSP9*, *CsCSP12*	*Conopomorpha sinensis*	λ-cyhalothrin	[52]
*PxCSP1*	*Plutella xylostella*	Indoxacarb	[53]
*PxCSP8*	*Plutella xylostella*	Permethrin	[47]
*BmorCSP1*, *BmorCSP2*, *BmorCSP4*, *BmorCSP7*, *BmorCSP10*, *BmorCSP9*, *BmorCSP13*, *BmorCSP11*, *BmorCSP12*, *BmorCSP15*, *BmorCSP19*, *BmorCSP14*, *BmorCSP17*, *BmorCSP20*	*Bombyx mori*	Avermectins	[46]
*AgosCSP5*, *AgosCSP4*, *AgosCSP6*	*Aphis gossypii*	Omethoate	[57]
*SlituCSP1*, *SlituCSP3*, *SlituCSP4*, *SlituCSP5*, *SlituCSP11*, *SlituCSP12*, *SlituCSP13*, *SlituCSP18*, *SlituCSP19*, *SlituCSP20*	*Spodoptera litura*	Chlorpyrifos	[55]
*SlituCSP2*, *SlituCSP3*, *SlituCSP4*, *SlituCSP5*, *SlituCSP6*, *SlituCSP11*, *SlituCSP12*, *SlituCSP13*, *SlituCSP20*	*Spodoptera litura*	Emamectin benzoate	[55]
*SlituCSP1*, *SlituCSP2*, *SlituCSP3*, *SlituCSP4*, *SlituCSP5*, *SlituCSP6*, *SlituCSP7*, *SlituCSP8*, *SlituCSP9*, *SlituCSP10*, *SlituCSP11*, *SlituCSP12*, *SlituCSP13*, *SlituCSP19*, *SlituCSP20*	*Spodoptera litura*	Fipronil	[55]
*SfruCSP1*, *SfruCSP2*	*Spodoptera frugiperda*	Spinetoram, Cypermethrin, Chlorantraniliprole, Chlorfenapyr, Chlorpyrifos, Indoxacarb	[34]
*SfruCSP22*	*Spodoptera frugiperda*	Cypermethrin	[34]
*unigene 3898*	*Ostrinia furnacalis*	Flubendiamide	[51]

It must be noted, however, that while CSP upregulation is noticed in the cases mentioned in Table 1 upon induction with sublethal doses of insecticides, this might not indicate a direct response to stimulus from exposure but can be attributed to several confounding factors. To fully validate these experimental results, one must take into account sample heterogeneity, variable experimental design, and different statistical testing among different studies. In several cases, CSP differential expression could be stochastic, and it does not necessarily imply causation towards resistance unless other lines of evidence point to the same direction. An interesting early counter-example comes from *B. tabaci*, where it was shown that while *BtabCSP1* is upregulated by thiamethoxam induction, it does not bind the insecticide with high affinity [45].

#### 3.1.2. Some *CSPs* Are Constitutively Overexpressed in Resistant Populations

While *CSP* upregulation upon treatment with sublethal doses of insecticides may provide some cues towards potential candidate proteins implicated in resistance, it is probably more informative to identify *CSPs* that are highly expressed in resistant strains, i.e., with an already-established mechanism of operational resistance in the field. Several examples of *CSPs* that are constitutively overexpressed in resistant strains have been identified in recent years. For instance, analysis of transcriptomic data of pyrethroid-resistant *Anopheles* mosquitoes showed that some *CSPs* are constitutively overexpressed in resistant populations compared with susceptible ones [56,59,60]. In two resistant field populations of *P. xylostella*, *PxCSP1* and *PxCSP3* were overexpressed compared to a control susceptible strain [53]. In comparative transcriptomic analysis of resistant vs. susceptible strains of *A. gossypii*, *CSP* genes were significantly upregulated in cyantraniliprole-resistant strains [61] and also in thiamethoxam- and spirotetramat-resistant ones [62]. In *R. padi*, the expression levels of *RpCSP6* and *RpCSP7* were significantly elevated in the resistant strain compared to that in the susceptible strain [49,50]. In *S. frugiperda*, transcription of two cuticular *CSPs* was considerably higher in the R strain than in the S strain, up to 9.0- and 8.0-fold, respectively [34]. A similar pattern was observed in resistant vs. susceptible strains of *N. lugens* [19].

On the other hand, CSP upregulation in resistant populations such as the ones listed above should be assessed considering also the heterogeneity of the relevant studies. Since the scope of each of these transcriptomic studies may vary, there are discrepancies in experimental design, sample size, and statistical testing that may lead to variation of reported effect sizes. Additional evidence is required to fully justify the involvement of CSPs in insecticide resistance.

#### 3.1.3. *CSP* Silencing May Lead to Increased Insecticide Toxicity

The potential involvement of CSPs in insecticide resistance is further supported by experimental evidence indicating that knockdown of *CSP* gene expression enhances insecticide toxicity. Several studies have provided such evidence, assessing insecticide-associated mortality following RNAi-induced silencing of several *CSPs* in different organisms (Table 2). RNA interference (RNAi) targeting the *SAP2* of *An. gambiae* in the resistant Tiassalé population resulted in significantly increased mortality rates in pyrethroid bioassays [56]. In the same research, RNAi of *CSP6* increased the sensitivity of the Tiassalé population to deltamethrin, although not to the same degree as *SAP2* knockdown.

Similar functional experiments were performed in different aphid species such as *A. gossypii* and *R. padi*. Suppression of *AgoCSP1*, *AgoCSP4*, and *AgoCSP5* transcription by RNAi significantly increased the sensitivity of resistant aphids to cyantraniliprole [61]. Similarly, the sensitivity of a thiamethoxam-resistant strain to thiamethoxam increased significantly with the silencing of *AgoCSP1* and *AgoCSP4*, while the sensitivity of a spirotetramat-resistant strain to spirotetramat increased significantly with the silencing of *AgoCSP4* [62]. RNAi experiments on *RpCSP4*, *RpCSP5*, *RpCSP6*, and *RpCSP10* increased *R. padi* mortality to imidacloprid, while RNAi to *RpCSP5* and *RpCSP6* increased mortality to β-cypermethrin [48]. Knockdown of *RpCSP7* increased aphid susceptibility to λ-cyhalothrin [63], while knockdown of *RpCSP6* significantly enhanced the susceptibility to deltamethrin [50]. In the same species, knockdown of *RpCSP4* and *RpCSP5* increased sensitivity to thiamethoxam [49].

In *P. xylostella*, RNAi targeting *PxCSP1* expression resulted in increased sensitivity of the moths to indoxacarb [53]. In *S. litura*, knockdown of *SlituCSP18*, followed by feeding with chlorpyrifos or fipronil, significantly decreased survival rates of male moths compared with controls [55]. In *S. frugiperda*, silencing of *SfruCSP1* and *SfruCSP2* increased larval susceptibility to chlorfenapyr, chlorpyrifos, and indoxacarb [34]. In *T. castaneum*, RNAi of *TcCSP10* increased the susceptibilities of the beetles to dichlorvos or carbofuran [54]. In the Asian citrus psyllid *Diaphorina citri*, RNAi of *DcitCSP8* increased the susceptibility to thiamethoxam, while injection of DcitCSP8 protein can restore the resistance [58]. Silencing of six *CSPs* from the brown planthopper *N. lugens* was also shown to increase susceptibility to imidacloprid [19].

While RNAi data (Table 2) provide certain indications for the association of CSPs with resistance, they cannot be considered ‘firm proof’ per se since there are inherent methodological shortcomings and limitations with this approach, including potential off-target effects, variability in relation to genetic background, failure to use appropriate controls, and the mere fact that the method is not standardized outside certain model species, since some species are more amenable to RNAi while in others, it is quite difficult to obtain meaningful inhibition of target genes in a reproducible way.

### 3.2. Functional Assays: In Vitro Insecticide Binding and Molecular Docking Simulations

Several studies have employed biochemical and biophysical methods to study binding of insecticides and other molecules to recombinant CSPs. These include competitive binding assays, usually using standard competition to a fluorescent N-phenyl-1-naphthylamine (1-NPN) probe or tryptophan fluorescence spectroscopy, as well as computational/molecular docking approaches using the small number of available CSP structures as templates for homology modelling. While certain CSPs were already known to strongly bind semiochemicals [24], binding of insecticides was demonstrated in several cases in recent years.

In *B. tabaci*, it was shown that BtabCSP1 does not bind thiamethoxam with high affinity and preferentially binds to linoleic acid, while BtabCSP2 and BtabCSP3 proteins are rather associated with completely different types of chemicals, indicating that some CSPs facilitate the transport of fatty acids while some others are tuned to much more volatile chemicals [45]. Multiple fluorescence spectra, thermodynamic methods, and molecular docking were used to study the interaction and the functional inhibition of imidacloprid to the recombinant CSP1 protein in the Asian honeybee, *Apis cerana*, indicating both imidacloprid binding and inhibition of binding of semiochemicals [64]. In vitro competitive binding assays in *An. gambiae* AgSAP2 show that it selectively binds pyrethroids [56].

Molecular docking and competitive binding assays indicated that certain CSPs from *A. gossypii* bind moderately with cyantraniliprole [60], while homology modeling, molecular docking, and dynamic simulation supported the interactions of AgoCSP5 with omethoate, imidacloprid, and cypermethrin and revealed a higher stability of AgosCSP5/insecticide complexes than AgosCSP5/semiochemical complexes [57]. Similarly, molecular docking and fluorescence competitive binding showed that RpCSP4 and RpCSP5 from *R. padi* had high binding affinity with thiamethoxam [49], while molecular docking predicted that there are hydrogen bonding sites which play key roles in the binding of RpCSP4, RpCSP5, RpCSP6, RpCSP7, and RpCSP10 with imidacloprid and β-cypermethrin [48]. The binding affinity of RpCSP6 to 24 commonly used insecticides was measured and seven key residues were found to steadily interact with deltamethrin, indicating their significance in the binding affinity to the insecticide [50].

In *C. sinensis*, CsCSP1, CsCSP2, CsCSP9, and CsCSP12 are capable of binding and transporting λ-cyhalothrin, while homology modeling and molecular docking analyses showed that the binding energy value of CsCSP1–12 to the insecticide was negative [52]. Using molecular dynamics simulations and site-directed mutation, it was found that indoxacarb forms a solid complex with PxCSP1 from *P. xylostella*, mainly through van der Waals and electrostatic interactions, and that the Lys100 side chain in PxCSP1 is the key factor for the high affinity of PxCSP1 to indoxacarb [53]. Binding experiments with recombinant SfruCSP1 and SfruCSP2 from *S. frugiperda* indicated that they can bind to chlorfenapyr, chlorpyrifos, and indoxacarb [34]. In *N. lugens*, the binding affinities of four CSPs to imidacloprid were confirmed through fluorescence competitive binding assays, and molecular docking indicated that their respective cavities are able to accommodate the insecticide [19].

Strong binding to insecticides has also been shown for CSPs which are expected to have a predominantly sensory function. In the swallowtail butterfly *Papillio xuthus*, PxutCSP19 is tuned to a monoterpenoid alcohol, linalool, which generally exists in host plants. However, PxutCSP19 is also capable of binding eight insecticides with stronger binding abilities compared to host odorants [65]. In *Dioryctria abietella*, four DabiCSPs with antenna-biased expression could bind three widely used insecticides (i.e., chlorpyrifos, phoxim, and chlorfenapyr), and DabiCSP1 was broadly tuned to twenty-seven plant-derived odors but also the three aforementioned insecticides and one herbicide with high affinities (Ki < 6.60 μM) [29]. Similarly, antennal SlitCSP6 could bind chlorpyrifos, emamectin benzoate, and fipronil while SlitCSP18 strongly binds chlorpyrifos and fipronil in *S. litura* [66]. For three CSPs enriched in the antenna and tarsi of the beetle *Rhaphuma horsfieldi*, binding assays showed that they were tuned differentially to insecticides but exhibited the highest affinities with hexaflumuron, chlorpyrifos, and rotenone [67].

In vitro binding assays have been extensively used in research associated with CSP function, but there are potential methodological shortcomings in this approach that should be also taken into account. While competition assays (most notably the 1-NPN assay) are frequently employed and work quite well for semiochemicals, they may be more problematic to standardize for chemical insecticides which are generally larger and more hydrophobic, indicating the need for more ‘orthogonal’ approaches. Molecular docking simulations have also been extensively used, but they are based on only a limited number of available CSP 3D structures.

### 3.3. In Vivo Evidence in Transgenic Insects

While binding assays in vitro and molecular dynamics simulations can be quite informative, they are not sufficient for in depth investigation of the potential involvement of CSPs to insecticide resistance, unless their findings are corroborated by research in vivo. A handful of studies have attempted to employ transgenic insects, and notably *Drosophila*, to perform ‘gain of function’ experiments by combining ectopic overexpression of candidate CSPs with toxicity bioassays against compounds in question.

These studies make use of a GAL4/UAS system [68], where expression of candidate *CSP* genes (cloned following Upstream Activating Sequences, UAS) in transgenic insects is activated by the transcription factor GAL4 expressed by relevant promoter ‘drivers’. In transgenic flies overexpressing *AgoCSP5* from *A. gossypii* with a heatshock-induced GAL4-driver (i.e., in all tissues), increased resistance to omethoate, imidacloprid, and cypermethrin was observed. While resistance ratio vs. controls was 2.61-fold for omethoate (n.d. for the other two compounds), molecular docking experiments suggested that imidacloprid is the strongest ligand among the three [57].

Transgenic *Drosophila* expressing three different *AgoCSPs* in the broad body (*Act*5C-GAL4 driver) or midgut (*Esg*-GAL4 driver) showed higher tolerance to cyantraniliprole than control flies with the same genetic background; *AgoCSP4* was more effective in broad body tissue, while *AgoCSP1* and *AgoCSP5* were more effective in the midgut (cyantraniliprole tolerance in contact toxicity assays for *AgoCSP1*, *AgoCSP4*, and *AgoCSP5* increased by 4.02-, 4.53-, and 1.66-fold, respectively, while the gastric toxicity assay showed 10.13-, 1.88-, and 9.01-fold higher tolerance, respectively [61].

*AgoCSP1* and *AgoCSP4* were also validated for resistance to thiamethoxam, α-cypermethrin, and spirotetramat. In the bioassays of thiamethoxam, the LD_50_ values of transgenic *Drosophila* expressing *AgoCSP1* and *AgoCSP4* increased by 2.80- and 6.76-fold, respectively, compared with those in the control in terms of contact toxicities while the LC_50_ values increased 1.37- and 1.23-fold in terms of gastric toxicities. For α-cypermethrin, the relevant figures were 1.06- and 5.76-fold for contact toxicities and 3.29- and 7.27-fold for gastric toxicity. The gastric toxicity of spirotetramat was significantly different between flies expressing *AgoCSP4* and control flies. These results indicate that overexpressed *AgoCSP1* and *AgoCSP4* can help *Drosophila* endure insecticide exposure [62].

Apart from the *Drosophila* model, the GAL4/UAS system was used for transgenic overexpression of *SAP2* in a population of susceptible mosquitoes [56]. While mosquitoes overexpressing *SAP2* remained susceptible, there was an observable reduction in mortality following permethrin exposure, indicating that overexpression can confer at least some resistance to pyrethroids and thus directly linking the function of this protein to insecticide resistance [56].

## 4. A Sequestration Resistance Mechanism and Possible Interactions

As described in detail, several lines of evidence point to a significant role of CSPs in insecticide resistance. However, the specific mechanisms by which CSPs may contribute to resistance need to be further investigated. So far, resistance mechanisms have been classified into four broad categories, namely behavioral resistance, penetration resistance, target-site resistance, and metabolic detoxification, the latter in connection with certain enzyme and transporter classes (cytochrome P450s, esterases, glutathione S-transferases, Uridine diphosphate (UDP)-glycosyltransferases, and ABC-transporters) that break down or wrap up (sequester) insecticide molecules before they reach their target sites. Since CSPs do not appear to have enzymatic functions, the most plausible hypothesis is that they act by sequestering insecticides. It is noteworthy that sequestration resistance has recently been elevated to a separate category of resistance mechanisms in some reviews of the field [69,70].

The ability of CSPs to solubilize and transport hydrophobic molecules along with their transcriptional overexpression in insect populations challenged with sublethal doses of insecticides or in resistant populations suggests that CSPs can confer resistance by binding to the insecticide molecule and potentially sequestering it, preventing it from reaching its target. It has been proposed that CSPs may act as buffers in gut tissues by sequestering and masking toxic insecticide molecules, which could then be discarded in the feces complexed to the proteins [5]. In resistant *An. gambiae*, SAP2 is highly concentrated in the legs of resistant mosquitoes, and since the legs are the point of entry in pyrethroid insecticides, it has been proposed that sequestration prevents pyrethroid action on the central nervous system [56]. A similar role is implicated for AgoCSPs sequestering and masking toxic insecticide molecules in *A. gossypii* [61] and certain CSPs in *N. lugens* [19], but also for the CSPs enriched in the antennae and tarsi of *R. horsfieldi* [67]. The two cuticle-enriched CSPs in *S. frugiperda* are also implicated in sequestration of insecticides, eventually reducing penetration efficiency [34].

**Figure 1:**
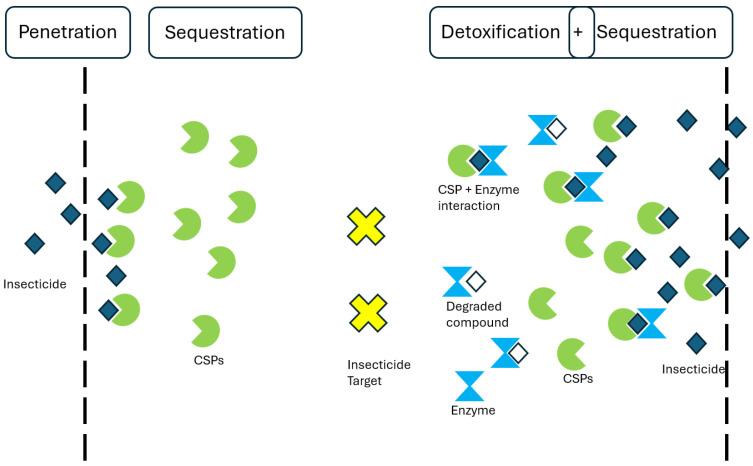
Potential model of CSP action towards insecticide resistance. CSPs may act by wrapping up insecticide compounds and preventing them from reaching their intended molecular targets, either at the point of entry (cuticle, legs, etc.) or at other tissues. Sequestration may work in combination with other mechanisms, presumably with detoxification enzymes which may facilitate degradation of toxic compounds bound to CSPs, providing a synergistic response by both sequestration and metabolic resistance acting simultaneously with enhanced efficiency.

While sequestration might prevent or delay insecticides from reaching their molecular targets, it most probably works together with other detoxification mechanisms such as metabolic or penetration resistance (Figure 1). CSPs could facilitate detoxification by making insecticide molecules accessible to degrading enzymes in a manner similar to the degradation of odorants by odorant-degrading enzymes. It is noteworthy that several enzyme classes responsible for odor degradation are also implicated in metabolic resistance to insecticides, like cytochrome P450s, carboxylesterases, and glutathione S-transferases [71]. While the precise molecular mechanism of such an interaction remains unclear, a ‘synergistic’ effect of multiple resistance mechanisms has been implicated in several cases [19,29,34,56].

It is noteworthy that coordinated expression patterns between *CSPs* and cytochrome P450 enzyme genes have been observed in response to insecticide treatment in several cases [19,46,47,51,56,60]. In the few available cases of in vivo verification of *CSP* overexpression leading to resistance, the observed resistance ratios indicate a rather limited effect of *CSP* overexpression alone [57,61,62]. As noted by Ingham et al. for transgenic *An. gambiae*, “*when SAP2 was overexpressed in an otherwise-susceptible background*, *the transgenic line was more resistant to pyrethroids*, *but the phenotype was less marked than the phenotypes found in the SAP2 knockdown lines*, *perhaps indicating that SAP2 acts in conjunction with other resistance mechanism(s) to provide an additive effect*” [56]. This is in agreement with the multiplicative outcome of combined vs. individual resistance mechanisms operating in the same genetic context that has been observed in pyrethroid resistance [72].

## 5. Concluding Remarks

While CSPs are known to play important roles in various physiological functions such as reproduction, semiochemical perception, and development, during the past decade, they have also been implicated to insecticide resistance, given their ability to bind to a wide range of hydrophobic molecules including insecticides, their upregulation following insecticide treatment, and the restoration of sensitivity to insecticides after silencing of *CSP* genes through RNAi. CSPs may take part in the sequestration and/or contribute to the detoxification of insecticides within the insect body, presumably in combination with other resistance mechanisms.

Currently, the understanding of CSPs-mediated resistance is far from complete. Most of the research in the field relies substantially on in vitro assessment of binding, molecular docking studies, and RNAi. Given certain methodological shortcomings of available competition assays, it will be desirable to broaden the assays and technologies used to assess interactions; approaches like microscale thermophoresis (MST), differential scanning fluorimetry (DSF), surface plasmon resonance (SPR), or other biophysical methods could be an option. Furthermore, while structural predictions can be useful, there are only a limited number of CSP 3D structures available. Novel approaches using AI like AlphaFold or technologies like Cryo-EM might offer new insights to define interactions. Finally, RNAi in non-model species (as in some models as well) may be quite troublesome to standardize and obtain comparable results.

In any case, however informative these methods can be, it is crucial to be able to perform in vivo studies that could experimentally verify hypotheses derived from in vitro work. Only a handful of such studies in transgenic *Drosophila* or target species are available at this point, and the whole concept of CSP-mediated resistance needs to be validated by in vivo results. Furthermore, it is crucial to investigate potential interactions between sequestration resistance mediated by CSPs and other resistance mechanisms operating in the same insect populations.

There is much more work to be done in this emerging field, not only to enhance our comprehension of the role and interactions of CSPs but also as a promising prospect for pest management. By elucidating the molecular mechanisms underlying CSP-mediated insecticide resistance, researchers can develop novel pest control strategies targeting these mechanisms. Employing functional and structural information on CSP-insecticide interactions, researchers can potentially design novel compounds that target these interactions and compete with the insecticides for binding to CSPs, enabling the generation of ‘resistance-breaking’ compounds that could act as synergists in insecticide formulations. This could enable much lower insecticide doses, inform control strategies, and could have significant potential impact on agriculture and public health.

## Figures and Tables

**Table 2:** Details of RNAi-mediated silencing of chemosensory proteins resulting in increased toxicity.

CSP Name	Organism	Insecticide (s)	Reference
*SAP2*	*Anopheles gambiae*	Deltamethrin, α-Cypermethrin, Permethrin	[56]
*AgoCSP1*, *AgoCSP4*	*Aphis gossypii*	Thiamethoxam	[62]
*AgoCSP4*	*Aphis gossypii*	Spirotetramat	[62]
*AgoCSP1*, *AgoCSP4*, *AgoCSP5*	*Aphis gossypii*	Cyantraniliprole	[61]
*RpCSP4*, *RpCSP5*	*Rhopalosiphum padi*	Thiamethoxam	[49]
*RpCSP7*	*Rhopalosiphum padi*	λ-cyalothrin	[63]
*RpCSP6*	*Rhopalosiphum padi*	Deltamethrin	[50]
*RpCSP4*, *RpCSP5*, *RpCSP6*, *RpCSP10*	*Rhopalosiphum padi*	Imidacloprid	[48]
*RpCSP4*, *RpCSP6*	*Rhopalosiphum padi*	β-cypermethrin	[48]
*PxCSP1*	*Plutella xylostella*	Indoxacarb	[53]
*SlituCSP18*	*Spodoptera litura*	Chlorpyrifos, Fipronil	[55]
*SfruCSP1*, *SfruCSP2*	*Spodoptera frugiperda*	Chlorfenapyr, Chlorpyrifos, Indoxacarb	[34]
*TcCSP10*	*Tribolium castaneum*	Dichlorvos, Carbofuran	[54]
*DcitCSP8*	*Diaphorina citri*	Thiamethoxam	[58]
*NluCSP2*, *NluCSP4*, *NluCSP5*, *NluCSP7*, *NluCSP12*, *NluCSP15*	*Nilaparvata lugens*	Imidacloprid	[19]
